# Kinetics effect and inhibition mechanism of Tartary buckwheat extracts on acrylamide in the asparagine-glucose system

**DOI:** 10.1016/j.fochx.2025.103158

**Published:** 2025-10-18

**Authors:** Wen Zhao, Yuchun Jing, Xiaoping Li, Xinzhong Hu, Lixia Wang

**Affiliations:** a College of Food Engineering and Nutritional Science, Shaanxi Normal University, Xi'an, Shaanxi 710062, China; b College of Life Sciences and Food Engineering, Shaanxi Xueqian Normal University, Xi'an 710100, China

**Keywords:** acrylamide reduction, Tartary buckwheat extracts, Maillard simulation system, kinetics study

## Abstract

This work investigated the kinetics effect and the inhibition mechanism of acrylamide (AA) formation by adding Tartary buckwheat extracts from Tartary buckwheat seeds (TB) and Tartary buckwheat sprouts (TBS) in the asparagine-glucose reaction system. TBS exhibited greater inhibitory activity against AA than TB, demonstrating inhibition rates of 47.95 % and 32.13 % at 1 mg/mL, respectively. Rutin, the main polyphenol of extracts, achieved the highest inhibition rate (64.69 %) at 10^−9^ mol/L. The Logistic-Exponential kinetic model was well fitted to the kinetic changes of AA in this study. In addition, the kinetics change of reaction products and oxidation-reduction potential indicated that Tartary buckwheat extracts and rutin might mitigate AA through three paths: 1) decrease the conversion of 3-aminopropionamide (3-APA) to AA, 2) reduce the formation of hydroxymethylfurfural (HMF), 3) enhance the elimination of AA by increasing the positive voltage of the model system.

## Highlights

• Tartary buckwheat extracts inhibit acrylamide formation in Maillard reaction.
• TBS showed higher inhibition rates (47.95 %) than TB (32.13 %) at 1 mg/mL.
• Rutin displayed the highest inhibition rate (64.69 %) at 10^−9^ mol/L.
• Logistics-exponential model best fits acrylamide kinetic changes.
• Inhibition pathways: 3-APA/HMF reduction and redox enhancement.

## 1 Introduction

In 2002, researchers found acrylamide (AA) in fried and baked starchy foods at levels over 500 times the WHO's acceptable limit in drinking water (Mottram et al., 2002). Studies have shown that AA could exhibit genotoxicity, carcinogenicity, neurotoxicity, and reproductive toxicity (Maan et al., 2022; Sarion et al., 2021). Due to its adverse effects, AA had been widely studied. It was recognized that Maillard reaction between asparagine and reducing sugar, such as glucose and fructose, was the major pathway for AA formation (Jing et al., 2019; Qi et al., 2018; Zhang & Zhang, 2008). The biosynthetic pathways of AA in thermally processed foods could be influenced by various factors, such as the concentration of precursor substances, pH, moisture content, and process parameters (Maan et al., 2022; Pesce et al., 2024). So the commonly used inhibition strategies for AA formation included selecting raw materials that contain lower levels of asparagine and reducing sugars, changing the cooking temperature and time, and adding the exogenous additives (Maan et al., 2022; Pesce et al., 2024).

Plant extracts contain high levels of phenolic acids and flavonoids, which give them antioxidant properties. Most studies indicated that plant extracts can mitigate AA levels. For instance, Morales et al. (2014) demonstrated that extracts from green tea, cinnamon, and oregano reduced AA levels in fried potato chips by 62 %, 39 %, and 17 %, respectively. Similarly, Verma and Yadav (2024) reported that plant extracts from curry leaves, mint, turmeric, cinnamon and cloves, when applied in conjunction with blanching, reduced AA content by 54.81 %, 50.76 %, 50.20 %, 45.52 %, and 44.27 %, respectively. Xu and An (2016) found that 0.1 mmol of p-coumaric acid reduced AA levels by 82.69 % when heated at 160 °C for 30 min in a Maillard model system. Adding p-coumaric acid to potato crisps also reduced AA by 66.2 % (Xu & An, 2016). These studies showed the value and practicality of using plant extracts in food applications for reducing the AA formation.

Tartary buckwheat (*Fagopyrum tataricum* (L.) Gaertn) has garnered significant attentions for its unique nutritional and medicinal properties. Tartary buckwheat seeds are rich in flavonoid compounds, with rutin being the most abundant flavonoid (Bhinder et al., 2022; Chen et al., 2022). Recent studies have shown that the germination of Tartary buckwheat seeds can significantly increase the content of active substances, such as rutin and chlorogenic acid (Bhinder et al., 2022; Chen et al., 2022). Research has demonstrated that the rutin content in germinated Tartary buckwheat seeds increases significantly compared to that in non-germinated Tartary buckwheat seeds (Bhinder et al., 2022; Kim et al., 2007; Ren & Sun, 2014). After 96 h of germination, free rutin levels can increase from 1811 mg/100 g to 1937 mg/100 g, enhancing the antioxidant capacity and free radical scavenging ability of the seeds (Bhinder et al., 2022). In the previous study (Jing et al., 2019), we found that addition of TB and TBS extracts (200 μL, each at a concentration of 1 mg/mL) can effectively reduce the formation of AA in bread. Crawford et al. (2019) also indicated flatbreads made with buckwheat flours had very low (< 10 μg/kg) levels of acrylamide. However, the inhibition kinetics and mechanism of buckwheat and its extract on AA formation is limited.

Change of AA is a dynamic process involving formation and elimination. The study of acrylamide kinetic models can further elucidate the dynamic process of its formation and elimination to provide a basis to explore the inhibitory mechanism of acrylamide formation. The most commonly used kinetic models of AA change are Logistic-Fermi kinetic models and Logistic-Exponential (or Logistic-Index) kinetic models (Corradini & Peleg, 2006; Nan et al., 2021; Zhang & Zhang, 2008). Nan et al. (2021) indicated that the Logistic-Index dynamic model and two successive simplified first-order kinetic models showed a strong fit to the changes of AA in the asparagine-glucose system with presence of glutathione and quercetin. Plant polyphenol extracts and polyphenolic compounds as antioxidants haves also been confirmed to modulate the kinetic processes involved in the formation and elimination of AA (Zhang & Zhang, 2008). Therefore, studying the impact of plant extracts on AA kinetics can reveal the mechanism by which these extracts inhibit AA formation. Kinetic and HPLC-MS/MS analyses revealed that quercetin suppresses AA formation by binding its decomposition products to Maillard reaction intermediates (Nan et al., 2021). In the past two decades, several critical intermediates, such as 3-APA, 3-oxypropionamide and HMF, have been identified to play an important role in the formation pathway of AA via the Maillard reaction (Liu et al., 2015; Maan et al., 2022). Concurrently, researchers have also found that plant polyphenolic compounds can modulate the conversion of intermediate products such as 3-APA and HMF to acrylamide, thereby inhibiting the formation of acrylamide. 3-APA has been considered an important direct precursor in the formation of AA, which can be transformed into AA after deamination (Cai et al., 2014; Qi et al., 2024; Wu et al., 2018). Lipophilic grape seed proanthocyanidin was found to decrease the content of acrylamide by reducing the content of sugar and 3-APA in potato-based products (Yu et al., 2020). Cheng et al. (2009) speculated that C-6 and C-8 of the A-ring in naringenin bonded with aldehyde groups in 3-oxopropanamide, which depleted 3-oxopropanamide and reduced the conversion from decarboxylated Schiff base to 3-APA, thus lowered the acrylamide formation rate. HMF, an important carbonyl intermediate in Maillard reactions, can react with asparagine to produce acrylamide (Qi et al., 2018; Zhu et al., 2022), while the condensation of epicatechin and HMF was one of the key steps leading to the inhibition of acrylamide in Maillard reactions model (Qi et al., 2018). Quercetin was found to trap HMF or its precursors to form corresponding adducts ultimately inhibiting HMF formation (Zhang & An, 2017). In addition, AA can be decomposed by free radicals generated during the Maillard reaction, resulting in the effective elimination of AA (Ou et al., 2010). Several studies have found that native or added catechin and its derivative, catechin quinone, can eliminate AA during thermal processing (Liu et al., 2023; Ou et al., 2008; Ou et al., 2010).

In conclusion, the use of exogenous polyphenols to inhibit AA formation during thermal processing has attracted widespread attention. However, to the best of our knowledge, there is still a lack of research on critical intermediates and the inhibitory mechanism of buckwheat extract on AA formation, and its kinetic model. Based on the literature and our research progress, we speculate that buckwheat extract may affect the level of AA by modulating the kinetics of AA and the conversion of the intermediate products to acrylamide in Maillard reaction. Therefore, this study aims to investigate the inhibition effects of Tartary buckwheat extracts on acrylamide formation in the Asn-Glc model and evaluate the dynamic process of formation and elimination of acrylamide by establishing kinetic models. The changes of intermediate (such as HMF and 3-APA), end product melanoidins and oxidation-reduction potential were also monitored in the model systems to better understand the inhibition mechanisms. The present study is expected to provide evidence for the application of Tartary buckwheat extracts in inhibiting AA formation in baked foods to improve its safety.

## 2 Material and methods

### 2.1 Chemicals and materials

Tartary buckwheat was sourced from Saixue (Dingbian County, Shaanxi Province, China). Asparagine, glucose, acrylamide (99 %), 3-APA (98 %) and HMF were commercially purchased from Jingbo Biotech (Xi'an, Shaanxi Province, China).

### 2.2 Preparation of TB and TBS extracts

Tartary buckwheat sprouts (TBS) were produced in accordance with the method outlined by Ling et al. (2018). Initially, shriveled seeds and impurities were removed from the Tartary buckwheat seeds (TB). Then, 50 g of the previously screened TB were rinsed with distilled water in a beaker and placed in a dark location at room temperature for 20 h. Following the soaking process, the TB were drained and transferred to a perforated plastic tray lined with two layers of sterile gauze. Subsequently, germination was conducted under the following conditions: the temperature was set to 20 °C, with 80 % relative humidity and a light-dark cycle of 12 h of light followed by 12 h of darkness. During the germination process, distilled water was sprayed three times a day at approximately 8 h intervals. On the tenth day of germination, the TBS were collected, measuring 13.3 cm of the average length and weighing approximately 278 g. Both TB (freeze-dried) and TBS samples were ground into powders. Equal amounts of TB powder and TBS powder were weighed and extracted using ultrasonic-assisted extraction with a 70 % (*v*/v) ethanol solution at a solid-to-liquid ratio of 1:10 (*w*/*v*). The extraction process was repeated three times, with each extraction lasting 30 min. The extraction liquids from each round were then combined to obtain TB extract and TBS extract. These extracts were subjected to freeze-drying, grinding, and stored at 4 °C for subsequent use and analysis.

### 2.3 Analysis of phenolic compounds in extracts

The TB and TBS extracts were determined in accordance with the method outlined by Jing et al. (2019) with some modifications. The chromatographic column was a Diamonsil C18 column (250 × 4.6 mm, 5 μm) from Agilent Technologies Co., Ltd., USA. And the separation process was conducted at 30 °C and a wavelength of 280 nm. The sample was eluted in a gradient with the mixture of formic acid, acetonitrile and purified water (0.1:5:94.9, v/v/v, solvent A) and the mixture of formic acid and acetonitrile (0.1:99.9, v/v, solvent B) at a flow rate of 1.0 mL/min. The gradient program was set as follows: 0–5 min, 95 %–87 % solvent A; 5–25 min, 87 %–70 % solvent A; 25–35 min, 70 % solvent A; 35–45 min, 70 %–95 % solvent A; and 45–50 min, 95 % solvent A.

### 2.4 Preparation of Maillard reaction simulated system

Maillard reaction simulated systems were established according to the method of Xu and An (2016) and Zhang and Zhang (2008), with some modifications. Tartary buckwheat extracts were prepared to a concentration solution of 10^−5^, 10^−4^, 10^−3^, 10^−2^, 10^−1^, 1 mg/mL, and rutin were prepared to a concentration solution of 10^−10^ to 10^−5^ mol/L. The equimolar asparagine (C_4_H_8_N_2_O_3_·H_2_O, MW 150.13) and glucose (C_6_H_12_O_6_·H_2_O, MW 198.17) powder (1.4 mmol, 487.6 g) were weighed and thoroughly ground together to ensure uniform mixing and sufficient surface reaction area. Subsequently, 200 μL of the extract or rutin solution (dissolved in ethanol) was introduced to reaction system. The reactants were then heated in an oven at 160 °C for 30 min. At the end of heating, the reaction products were rapidly cooled in an ice bath to stop any further reaction. The final reaction products were extracted using 20 mL of a 0.1 % formic acid aqueous solution. After thorough stirring on a magnetic stirrer for 30 min, the solutions were centrifuged at 4000 rpm for 10 min. Then the clear supernatants were filtered through 0.45 μm filters and stored at 4 °C for acrylamide analysis.

### 2.5 Determination of acrylamide

Determination of acrylamide were established according to the method of Shi et al. (2019), with some modifications. Acrylamide were analyzed on the U-3000 HPLC system. Ten microliters of the sample solution were injected onto a Diamonsil C18 column (250 × 4.6 mm, 5 μm) from Agilent Technologies Co., Ltd., USA. The column temperature was maintained at 30 °C, while the ultraviolet detector was calibrated to a wavelength of 210 nm. The sample was isocratic elution with the mixture of methanol and water (5:95, *v*/v) at a flow rate of 0.6 mL/min.

### 2.6 Kinetic study of AA by adding extracts

The equimolar quantities of asparagine and glucose (1.4 mmol of each component) underwent metrological quantification followed by particle size reduction through ground together. The optimal concentration of extracts/rutin that demonstrated the most significant reduction in AA formation was determined in the kinetic study. Subsequently, 200 μL of either the extracts or rutin was introduced to each system. The reactants were simultaneously heated in an oven at 160 °C for durations of 0, 5, 10, 15, 20, 25, 30, 35, 40, 45 and 50 min. Following the heating process, the resulting reaction products were rapidly cooled in an ice-water. The cooled products were prepared for the analysis of AA after extraction.

### 2.7 Kinetic models

#### 2.7.1 Logistic-Fermi model

Kinetic models were established using Origin 9.0 software. For the Logistic-Fermi model (Zhang & Zhang, 2008), C_f_(*t*) represents the variation in AA concentration during the formation phase, and *C*_e_(*t*) represents the variation in AA concentration during the elimination phase. The change of AA content in the whole reaction stage (*C*(*t*)) was represented by the product of C_f_(*t*) and *C*_e_(*t*).

$$\begin{array}{l} C_{f}\left(t\right)=\frac{a}{1+\exp\left[k_{f}\left(t_{f}-t\right)\right]}-\frac{a}{1+\exp\left(k_{f}\times t_{f}\right)}\\ C_{e}\left(t\right)=\frac{1}{1+\exp\left[k_{e}\left(t-t_{e}\right)\right]}\\ C\left(t\right)=C_{f}\left(t\right)\times C_{e}\left(t\right) \end{array}$$

The parameter *a* represents the temperature dependent coefficient related to AA initial concentration. The parameter *k*_f_ and *k*_e_ represent temperature-related steepness parameters in the process of formation and elimination, respectively. The parameter *t*_f_ and *t*_e_ respectively represent the time at the inflection point related to temperature in the process of formation and elimination.

#### 2.7.2 Logistic-exponential model

For the Logistic-Exponential model (Zhang & Zhang, 2008), the process of AA elimination is characterized by a straightforward exponential function. The change of AA content in the whole reaction stage (*C*(*t*)) could also be expressed by the product of C_f_(*t*) and *C*_e_(*t*).

$$\begin{array}{l} C_{f}\left(t\right)=\frac{a}{1+\exp\left[k_{f}\left(t_{f}-t\right)\right]}-\frac{a}{1+\exp\left(k_{f}\times t_{f}\right)}\\ C_{e}\left(t\right)=\exp\left(-\frac{t}{p}\right)\\ C\left(t\right)=C_{f}\left(t\right)\times C_{e}\left(t\right) \end{array}$$

The parameters of C_f_(*t*) have the same meaning as Logistic-Fermi model. The parameter *p* is the characteristic time of AA in the elimination process.

### 2.8 Kinetic study of melanoidins by addition of extracts

Determination of melanoidins were established according to the method of Knol et al. (2005), with some modifications. Ultrapure water was added to the final reaction product of the simulation system and sonicated for 30 min, and finally added water to make up to 50 mL. The mixture was detected at 470 nm. The concentration of melanoidins in the sample was calculated by Lambert-Beer equation, and the extinction coefficient of melanoidins was 282 L/ (mol·cm).

### 2.9 Effect of extracts on the formation of acrylamide from 3-APA

The mixed reaction substrates containing 100 μmol of 3-APA and 200 μL of extracts (1 mg/mL) or rutin (10^−9^ mol/L) was oven-heated at 160 °C for 5, 10, 15, 20, 25, 30 min, respectively. The method for determining AA content was the same as in 2.5.

### 2.10 Effects of extracts on HMF in Maillard reaction system

The reaction products of the Maillard reaction system with different reaction times (5, 10, 15, 20, 25, 30 min) were extracted using 20 mL of ultrapure water with magnetic stirring for 30 min. The samples were analyzed using an HPLC system equipped with a Diamonsil C18 column (250 × 4.6 mm, 5 μm) according to the method described by Gokmen et al. (2012) with some modifications. The column temperature was 40 °C. The separation was performed using a mobile phase of 0.1 % aqueous acetic acid and acetonitrile (90:10, *v*/v) at a flow rate of 1.0 mL/min, with UV detection at 285 nm and an injection volume of 10 μL (Gokmen et al., 2012).

### 2.11 Effect of extracts on oxidation-reduction potential in the Maillard reaction system

Determination of on oxidation-reduction potential were established according to the method of Rizzi (2003), with some modifications. 20 mL of ultrapure water was added to the reaction products of Maillard reaction system with different reaction time, and its oxidation-reduction potential was determined at various reaction times using a pH detector equipped with an oxidation-reduction potential (ORP) electrode.

### 2.12 Effect of extracts on elimination of acrylamide

1 mL of AA standard solutions at a concentration of 10 mg/L and 100 mg/L were added to a test tube respectively. An aliquot of extracts (1 mg/mL, 200 μL) was added to each treatment group, and an equal volume of rutin (10^−9^ mol/L) was added to the rutin control group. The solutions were heated in an oven at 160 °C for 30 min.

### 2.13 Statistical analysis

The statistical analyses were conducted using SPSS software version 18. To identify significant differences between samples, one-way ANOVA tests and Duncan test was implemented, with a significance level set at *P* < 0.05. Pearson correlation test is used for correlation analysis. Construct a kinetic model using Origin 9.0 software and evaluate the model using pseudo-correlation coefficients (pseudo-R^2^):

$$\text{pseudp}‐R^{2}=1-\frac{{\mathrm{SS}}_{\text{residual}}}{{\mathrm{SS}}_{\text{corrected}}}$$

## 3 Results and discussion

### 3.1 The polyphenolic component of the extracts

**Fig. 1:**
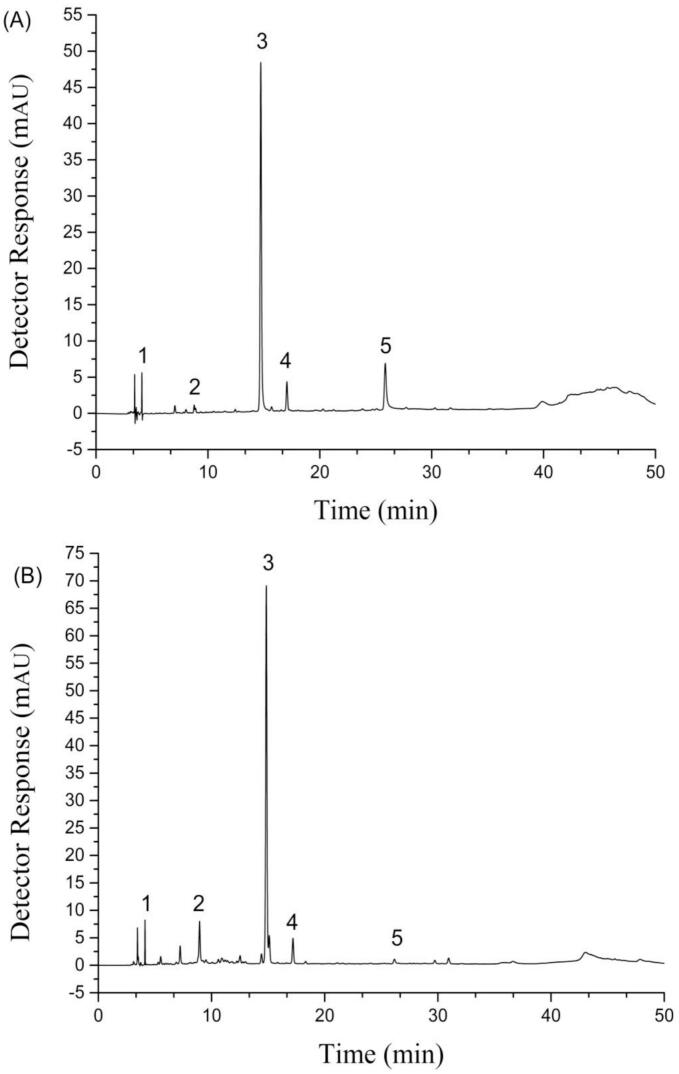
HPLC chromatograms of TB (A) and TBS (B) extracts. Compounds 1–5: Gallic Acid, Chlorogenic Acid, Rutin, Ferulic Acid, and Quercetin. Note: TB (Tartary buckwheat seeds), TBS (Tartary buckwheat sprouts).

Chromatograms of the extracts are displayed in Fig. 1 and the contents of the polyphenol components are presented in Table 1. Five polyphenolic compounds were identified in the extracts of TB and TBS, including rutin, gallic acid, quercetin, chlorogenic acid, and ferulic acid. And rutin was the most abundant phenolic compound with a concentration of 114.89 mg/g DW in TB and 152.37 mg/g DW in TBS. Several other compounds were present in lower concentrations in two extracts. The current findings align with those of previous studies conducted by other researchers. Ke et al. (2023) reported that rutin and quercetin are the main flavonoids in TB. Kim et al. (2007) found the content of rutin and chlorogenic acid in TBS was 52.95 mg/g and 4.3 mg/g respectively. After 20 h of germination, the content of rutin reached 15.8 mg/100 g DW, and after ten days of germination the rutin content reached 43.7 mg/100 g FW (Koyama et al., 2013). Bhinder et al. (2022) found that when TB germinated for 72 h, the content of rutin increased from 1811 mg/100 g DW (0 h) to 2129 mg/100 g DW (72 h) and the quercetin content increased from 329 mg/100 g DW (0 h) to 385 mg/100 g DW, but changed to 237 mg/100 g DW (96 h). Ren and Sun (2014) indicated that rutin in the buckwheat sprouts gradually increased and reached the maximum levels on days 9 of germination, but quercetin in TB decreased from day 5 and became undetectable on day 7. The change trend in quercetin content in these studies is consistent with our research result. The discrepancy of polyphenol content in buckwheat sprout may arise from Tartary buckwheat varieties and differences in germination conditions and day. Kim et al. (2007) found that phenolic compounds in the edible portions of TBS increased linearly during plant growth at 6–10 day, while rutin was the major flavonoid with mean 20 and 37 mg/g DW in ‘HokkaiT8’ and ‘HokkaiT10’, respectively. However, the contents of quercetin were only 0.32 mg/g DW (‘HokkaiT8’) and 0.12 mg/g DW (‘HokkaiT10’) (Kim et al., 2007).

**Table 1:** The contents of polyphenol components in Tartary buckwheat extracts(mg/g DW).

Extracts	Gallic acid	Chlorogenic acid	Rutin	Ferulic acid	Quercetin
TB	1.09 ± 0.06	0.09 ± 0.03	114.89 ± 4.36	1.47 ± 0.30	13.72 ± 1.53
TBS	0.52 ± 0.18	6.30 ± 1.35	152.37 ± 5.49	1.66 ± 0.62	0.13 ± 0.05

Data are expressed as means ± SD (*n* = 3).

### 3.2 Inhibitory effect of extracts on AA formation

**Fig. 2:**
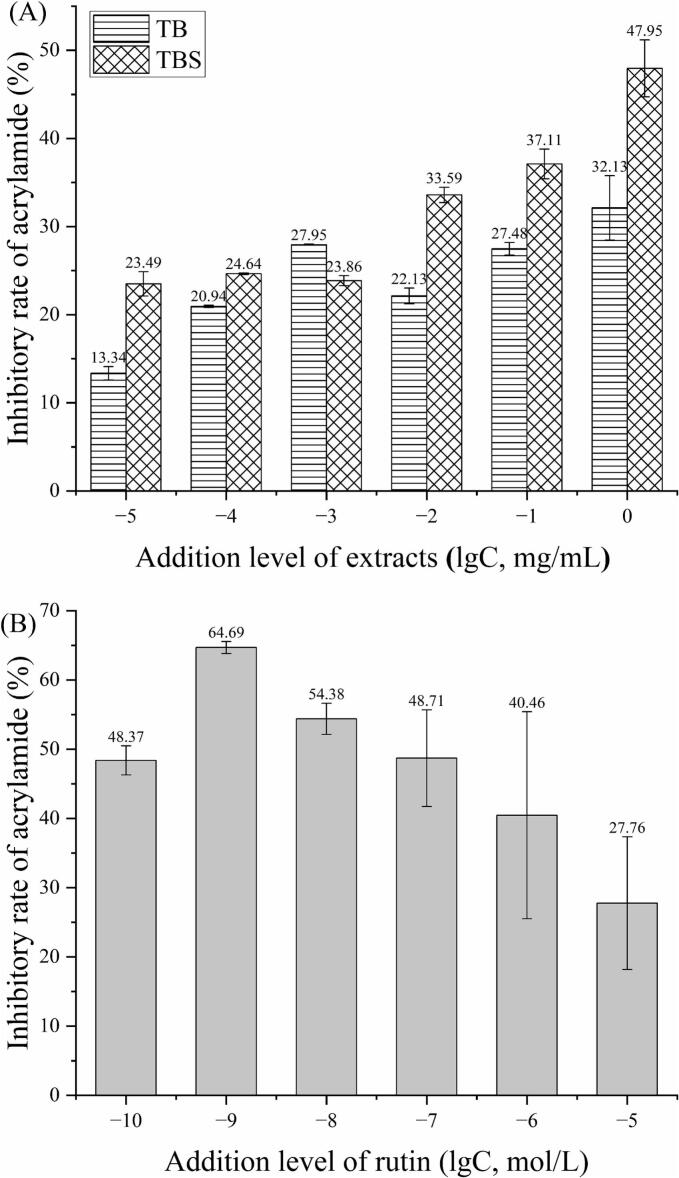
The inhibitory effect of TB and TBS extracts (A) and rutin (B) on acrylamide formation. The results are presented as the average ± SD (*n* = 3).

Effect of different addition levels of extracts on the reduction rate of AA was illustrated in Fig. 2 (A). The results indicated that Tartary buckwheat extracts inhibited AA formation at concentrations ranging from 10^−5^ to 1 mg/mL, with a reduction rate from 13.34 % to 47.95 %. The reduction rate of AA rose with the concentration increase of extracts. When the concentration of extracts (TB, TBS) was 1 mg/mL, the reduction rates of AA reached the maximum values of 32.13 % (TB) and 47.95 % (TBS), respectively. And reduction rate of TBS extracts on AA formation was higher than that of TB extracts. Present results are corresponded to those of our previous reports (Jing et al., 2019), in which a concentration-dependent relationship was observed between the reduction rate of AA in bread and the level of buckwheat extracts addition. It is similar to the results of Xue et al. (2022) who found the inhibition rate of AA was positively correlated with curcumin concentration, indicating that higher curcumin concentrations lead to stronger inhibitory effects on AA formation. The higher AA reduction rate observed in TBS extracts than in TB may be due to an increase in the quantity of active ingredients, such as phenolic compounds and flavonoids, present in the extract. As shown in Fig. 1, there were significant differences in phenolic compounds composition and content between the two buckwheat extracts. Compared with TB extracts, the content of chlorogenic acid, rutin and ferulic acid in TBS extracts increased, while the content of quercetin and gallic acid decreased. The diversity in polyphenol content and composition may lead to significant differences in their inhibition rates of acrylamide. Zhu et al. (2009) found that among 35 plants aqueous extracts at concentration of 0.1 mg/mL, 34 can reduce the content of acrylamide in an asparagine/glucose model system, with a reduction rate ranging from 11 % to 75 %. Similarly, among the 11 polyphenols, 9 can reduce the content of acrylamide (Zhu et al., 2009). Liu et al. (2015) summarized that the polyphenols affected the acrylamide formation during heating, depending on their structure, concentrations, and antioxidant capacity, as well as reaction condition.

To further explore the possible inhibition mechanism of the extracts on AA formation, a primary flavonoid (rutin), which is abundant in Tartary buckwheat extracts, was used to confirm the ability of polyphenols to reduce AA levels. The results presented in Fig. 2 (B) revealed that the addition of rutin can inhibit the formation of AA and the inhibition rate of AA by rutin initially increases and then decreases. Furthermore, the reduction rate of AA by rutin reached a maximum value at a concentration of 10^−9^ mol/L (64.69 %), which is consistent with a previous report (Cheng et al., 2013). They determined that the optimal concentration of six flavonols (rutin, kaempferol, kaempferol-3-O-glucoside, quercetin, quercetin-3-O-glucoside, myricetin) to inhibit AA formation was 1 × 10^−9^ mol/L (Cheng et al., 2013). However, Zhu et al. (2009) reported that the reduction rate of acrylamide by rutin at a concentration of 1 mmol/mL in an asparagine/glucose model system is 49.4 %. In recent years, research on the impact of rutin on AA has mainly focused on the mitigating effects of rutin on AA induced damage to the brain, kidneys, and liver. Studies have confirmed that these protective effects are closely related to rutin's antioxidant and anti-inflammatory activities (Uthra et al., 2022; Zhang et al., 2023). The Maillard reaction generates free radicals and oxidative intermediates (such as 3-aminopropionamide), which are considered key factors in the formation of acrylamide (Nagpal et al., 2022). Rutin may inhibit acrylamide formation by scavenging free radicals or reducing oxidative intermediates.

Based on current findings, there remains some discrepancy between the optimal concentrations of the extract and rutin in inhibiting acrylamide formation in simulated systems. This may be attributed to the presence of multiple polyphenolic components in the extracts. Although rutin is the most abundant, the extracts also contain a certain amount of gallic acid, chlorogenic acid, ferulic acid and quercetin (As showed in Table 1). Research has demonstrated that different types of polyphenols vary in their effective concentrations and efficacy in inhibiting acrylamide formation due to structural differences (Cheng et al., 2015). The maximum inhibition rates for AA were 51.38 % with 10^−1^ mol/L quercetin (Nan et al., 2021). Several studies revealed that chlorogenic acid at low level (30 μmol/100g) or at high level (1 mmol/mL) decreased acrylamide formation both in biscuits and asparagine/glucose reaction model (Oral et al., 2014; Zhu et al., 2009), however, they also could increase the formation of AA during thermal processing at moderate concentration (50 μmol/mL) (Cai et al., 2014). Another study has also reported that silymarin may promote acrylamide formation (Liu et al., 2015). The specific effect depends on the type and concentration of the antioxidant. In addition, multiple complex components in the extracts may interact with each other, affecting their inhibitory on AA. Therefore, polyphenolic compounds and plant extracts are only beneficial in inhibiting acrylamide formation within an appropriate concentration range.

### 3.3 Effect of extracts on the AA formation kinetic curves

**Fig. 3:**
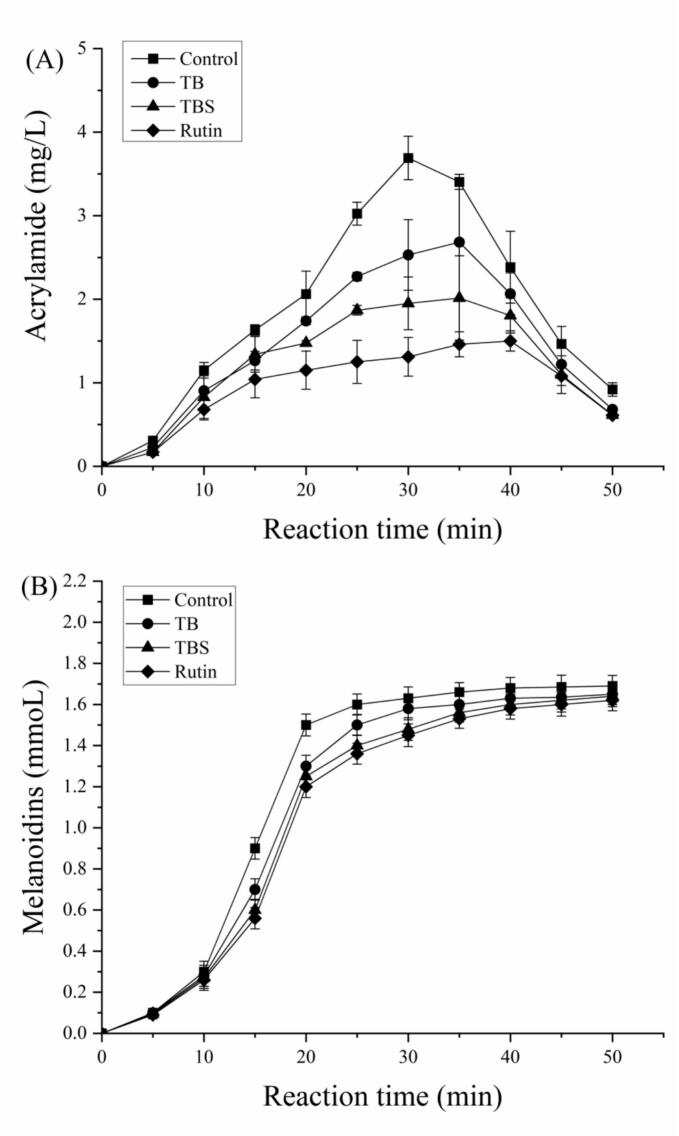
Kinetic curves of acrylamide (A) and melanoidins (B) in the control and the experimental groups (addition of TB and TBS extracts and rutin) in simulated system.

The kinetic curves of AA generation at different heat treatment times in the control and test groups in simulated system were presented in Fig. 3 (A). The optimized addition levels of extracts (1 mg/mL) and rutin (10^−9^ mol/L), were utilized for the kinetic study. As shown in Fig. 3 (A), the level of AA increased rapidly in the initial stage, and then began to decrease after the AA level reaching the maximum value, which indicated that the formation and elimination of AA in the simulation systems was a dynamic process. The concentration of AA in all group reached its maximum value within 30 to 40 min of heat treatment. Compared with the control group, the level of AA at different reaction time was decreased by the addition of extracts and rutin. The maximum level of AA treated with Tartary buckwheat extracts and rutin was markedly mitigated compared with the control group. Furthermore, the rutin treatment group exhibited the highest mitigation efficiency, followed by the TBS treatment group. As rutin was the main polyphenol components in TBS extracts, these results revealed that the reduction effect of AA by the addition of extracts might be related to the rutin contained in the extracts. In addition, compared with the control group, the time when the AA level reached the maximum value was delayed by addition of extracts and rutin. Time of reaching the maximum value of AA level in control group, TB extracts groups and rutin treatment group was 30, 35 and 40 min, respectively. When the heating duration surpassed 40 min, a markedly decrease in AA levels was detected in both the control and treatment groups. Meanwhile it can be seen in AA elimination-predominant stage, the content of AA in the treatment group also were lower than that in the control group, but the difference is not significant in the later stage of elimination. These results indicated that the reduction effect of Tartary buckwheat extracts and rutin on AA primarily occurs during the formation-predominant stage, rather than during the elimination-predominant stage. This was due to the antioxidant properties and thermal stability of Tartary buckwheat extracts and rutin. Our previous reports have demonstrated that the inhibitory effect of Tartary buckwheat extracts on the formation of AA was associated with their antioxidant activity (Jing et al., 2019). And the antioxidant of Tartary buckwheat extracts might be decreased with extended heating durations (Zhang et al., 2010). Meanwhile, the antioxidant activity of rutin was found to decrease when exposed to elevated temperatures or extended heating durations (Chaaban et al., 2017).

### 3.4 Kinetic model analysis

Firstly, the Logistic-Fermi kinetic model was established for the purpose of estimating the dynamic behavior of AA change with and without extracts and rutin in the asparagine-glucose system. Parameters and pseudo-R^2^ values of the Logistic-Fermi kinetic models were presented in Table 2. The Logistic-Fermi model comprised five kinetic parameters and exhibited considerable flexibility (Zhang & Zhang, 2008). The pseudo-R^2^ values (> 0.93) indicated a strong correlation between the prediction values calculated from the Logistic-Fermi model and the trial values of AA. However, the parameter *a* displayed high variability, with large standard deviations observed in both control and TB-treated groups. And the recommended values of the parameter *t*_e_ were puzzling because *t*_e_ were obviously inconsistent with the inflection point time previously obtained from the kinetic curve. Therefore, Logistic-Fermi is not an ideal model in the present research.

**Table 2:** Logistic-Fermi models parameters and pseudo-R^2^ for acrylamide in asparagine-glucose system.

System	*a* (μg/mL)	*k*_f_ (min^−1^)	*t*_f_ (min)	*k*_e_ (min^−1^)	*t*_e_ (min)	pseudo-R^2^
Control	1583.96 ± 625.64	0.05 ± 0.03	143.97 ± 99.87	0.20 ± 0.05	35.34 ± 2.39	0.9807
TB	1644.15 ± 1094.01	0.22 ± 0.02	39.21 ± 1.54	0.17 ± 0.02	5.036 ± 3.97	0.9818
TBS	10,009.21 ± 1809.71	0.25 ± 0.01	44.13 ± 1.18	0.23 ± 0.03	8.58 ± 1.82	0.9827
Rutin	321,709.78 ± 1556.35	0.32 ± 0.04	47.57 ± 0.63	0.31 ± 0.04	7.95 ± 1.05	0.9353

**Fig. 4:**
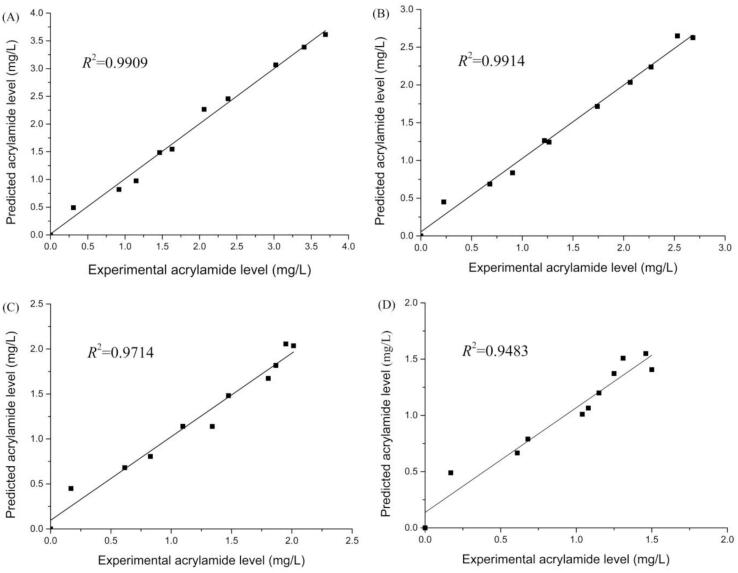
The correlation between predicted and experimental acrylamide level. (A) Control, (B) TB, (C) TBS, (D) Rutin. Note: TB (Tartary buckwheat seeds), TBS (Tartary buckwheat sprouts).

Secondly, the Logistic-Exponential model was employed in the current research. The parameter *a* was linked to the original concentration of the precursor. The parameter *k*_f_ denoted the AA formation rate. The *t*_f_ represents the time at which the concentration profile of AA reaches its inflection point. Parameters of the Logistic-Exponential kinetic models were presented in Table 3. Pseudo-R^2^ values (> 0.93) indicated good model fit for both control and treated groups. The kinetic parameter *k*_f_ of all treated groups with TBS extracts and rutin were markedly lower than that of the control group (*P* < 0.05). And the kinetic parameter *t*_f_ for treated group with TBS extracts or rutin showed a statistically significant increase in comparison with the corresponding value observed in the control system (*P* < 0.05). Thus, these results reveal that TBS extracts and rutin could limit AA formation during the preliminary reaction process. TBS extracts and rutin have also been observed to delay reactions time of AA levels reached their max. However, there was no significant difference compared to the control group (*P* > 0.05) for the parameter *p*, which represented the degradation status of AA. This indicated that the addition of extracts or rutin did not significantly reduce the AA content during the elimination dynamic phase. Scatter plots of relationship between AA predicted values and experimental values in simulated systems were shown in Fig. 4. The data demonstrated a strong correlation between the predicted and experimental AA values in both the control model and the treatment model, which indicated the good fitness of Logistic-Exponential model. In a word, the Logistic-Exponential model could be utilized to investigate the dynamic behavior of AA in present study. The previous researches (Nan et al., 2021; Zhang et al., 2015; Zhang & Zhang, 2008) are consistent with our findings. Zhang and Zhang (2008) investigated the effects of antioxidants and green tea extracts on the dynamic behavior of AA in a low-moisture system and the logistic-exponential model was selected among three kinetic model candidates. They found the additive showed significant mitigation on AA formation but not on AA elimination. Zhang et al. (2015) indicated that flavonoid treatment reduced AA levels by 51.0–59.9 % during the initial 4 min of heating. This effect primarily influences the generation stage of AA, with minimal impact on the elimination stage (Zhang et al., 2015). Nan et al. (2021) found that the Logistic-Index dynamic model and two successive simplified first-order kinetic models showed a strong fit to the changes of AA with addition of glutathione and quercetin in the asparagine-glucose (Asn-Glc) system. They also revealed that the kinetic results reflected that quercetin might mitigate AA through the binding reaction of decomposition products and Maillard intermediates (Nan et al., 2021).

**Table 3:** Logistic-Exponential models parameters and pseudo-R^2^ for acrylamide in asparagine-glucose system.

System	*a* (μg/mL)	k_f_ (min^−1^)	t_f_ (min)	*p* (min)	pseudo-R^2^
Control	580.66 ± 172.56	0.22 ± 0.007	33.77 ± 0.42	7.66 ± 0.85	0.9870
TB	850.99 ± 128.46	0.20 ± 0.005	36.32 ± 0.38	7.08 ± 0.81	0.9869
TBS	503.93 ± 99.26	0.18 ± 0.006	37.64 ± 0.99	7.69 ± 1.77	0.9560
Rutin	1748.91 ± 210.53	0.18 ± 0.004	43.32 ± 2.73	6.57 ± 1.93	0.9338

Data for Parameters of the Logistic-Fermi models are expressed as means ± SD (*n* = 3).

Data for Parameters of the Logistic-Exponential models are expressed as means ± SD (n = 3). Different letters within the same column indicate significant difference (*P* < 0.05).

### 3.5 Effect of extracts on the melanoidins formation kinetic curves

Melanoidins were a primary product of the Maillard reaction and serves as an indicator of Maillard reaction level (Iriondo-DeHond et al., 2020). The kinetics of the formation of melanoidins are shown in Fig. 3 (B). In all treatment groups, the content of melanoidins increased with the reaction time, gradually stabilizing after 30 min. Adding Tartary buckwheat extracts or rutin at the early stage of the reaction reduced melanoidin formation. The order of melanoidin content from high to low was as follows: control group, TB group, TBS group and rutin treatment group. This indicated that the addition of Tartary buckwheat extracts decreased the extent of the Maillard reaction, with the rutin group showing the greatest reduction. Reduction rate of the TBS group was higher than that of TB group. Our findings were consistent with the results reported by Zhang et al. (2015), who indicated that flavonoids may significantly reduce the development of melanoidins during the steep growth stage, but have a mild effect during the gentle increase stage.

### 3.6 Effect of extracts on the conversion of 3-APA to AA

**Fig. 5:**
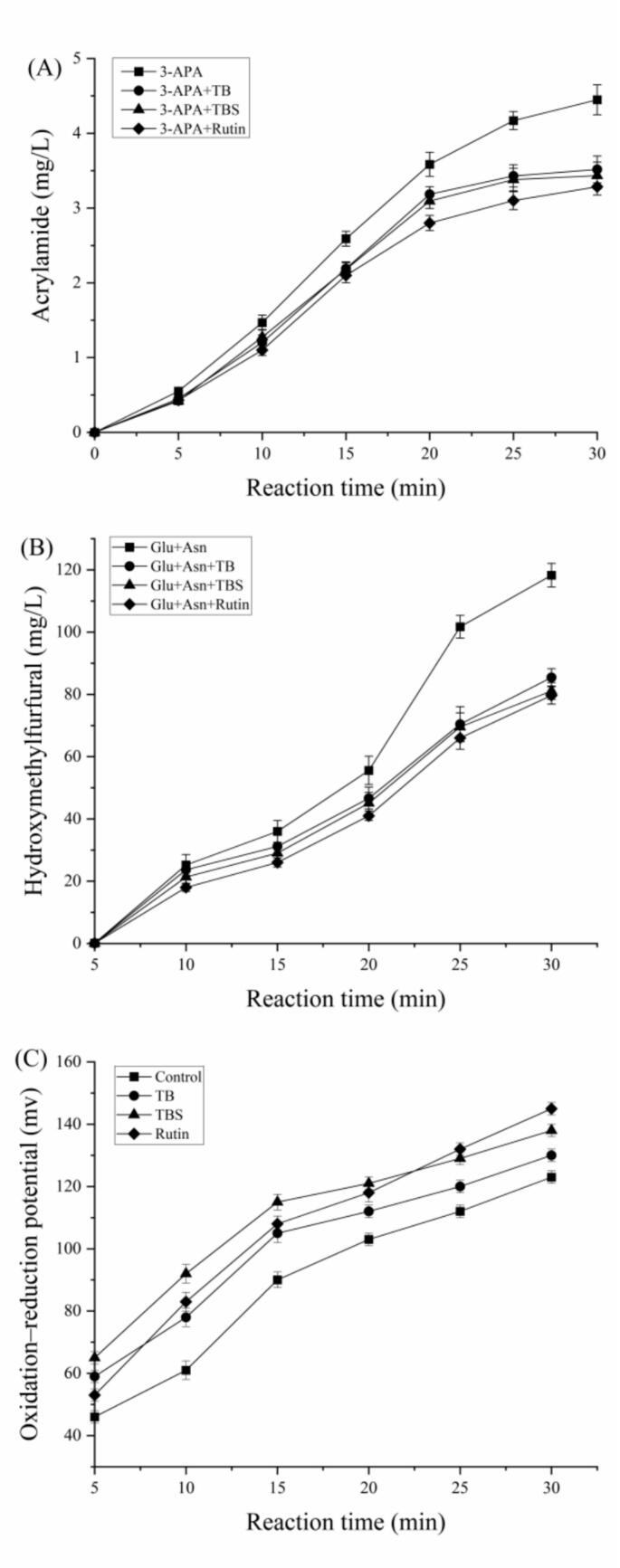
The formation of acrylamide from 3-APA with TB, TBS, and rutin at different heating times (A); Changes in HMF (B) and Oxidation-Reduction Potential (C) in the Asn-Glc simulated system with TB, TBS, and rutin at different heating times.

3-APA is considered a precursor for the formation of AA, which can be transformed into AA after deamination (Cai et al., 2014; Qi et al., 2024; Wu et al., 2018). To investigate the inhibitory effect of buckwheat extracts on the conversion of 3-APA to AA, this study established three experimental groups: 3-APA + TB, 3-APA + TBS, and 3-APA + rutin, with 3-APA as the blank control. All of groups were oven-heated at 160 °C for 5, 10, 15, 20, 25, 30 min, respectively. The changes in AA levels in each group were measured. As shown in Fig. 5 (A), as the heating time increased, the content of AA in all groups showed a concomitant increase trend. It can also be seen that AA formation from 3-APA with addition of extracts or rutin groups was lower than the control group. The mitigation efficiency of rutin group on the conversion of 3-APA to AA was the largest, followed by TBS group. These results indicated that 3-APA formed AA under heating conditions, while the extracts and rutin can reduce the formation of AA by inhibiting the conversion of 3-APA to AA. Several documents found that 3-APA forms AA under heating conditions (Cai et al., 2014; Zamora et al., 2025), which are consistent with our current results. However, there is still some controversy over the effect of polyphenols on the conversion of 3-APA to AA. The quinone derivative of chlorogenic acid could inhibit the formation of AA by preventing the transformation of 3-APA into AA (Cai et al., 2014). It can also be found from a recent document that hydroxytyrosol quinone and caffeic acid quinone also reduced the conversion of 3-APA to AA (Zamora et al., 2025). However, high concentrations of chlorogenic acid promoted AA formation, which may involve its ability to reduce the activation energy for the conversion of 3-APA to AA and increase deamination reaction of 3-APA (Cai et al., 2014). Zhang and Zhang (2008) indicated that the multiple phenolic hydroxyl groups in both homoorientin and EGCG act as hydrogen donors in the quinine-amine interaction between flavonoids and 3-APA, blocking the acrylamide formation pathway. However, they also proposed that polyphenols might reduce the acrylamide formation through oxidation to form quinones, which could react with 3-APA via quinone-amine interaction (Zhang & Zhang, 2008). The mechanism by which rutin reduces the conversion of 3-APA to AA is currently uncertain, and further research is needed to determine whether it is caused by phenolic hydroxyl groups or oxidation during the heat treatment. The difference in the efficiency of extract and rutin in affecting the formation of AA from 3-APA may be attributed to the presence of multiple other polyphenolic components, such as chlorogenic acid or quercetin in TB and TBS extracts. These coexisting components may exhibit complex interactions, including synergistic or antagonistic effects, which can influence the conversion efficiency.

### 3.7 Effects of extracts on the formation of HMF

HMF was an important intermediate to form AA in the Maillard reaction, and it reacts with asparagine producing more AA than glucose reacting with asparagine (Gokmen et al., 2012). This suggested that HMF production promoted AA formation in the Maillard reaction system (Gokmen et al., 2012). So the effect of extracts and rutin on HMF content in the Maillard reaction system was ascertained. The HMF content were showed in Fig. 5 (B). It can be seen that HMF content increased with the increase of heating time in all groups (Fig. 5 (B)). And the HMF content treated with extracts or rutin was much lower than that of the control, especially when the heating time exceeded 20 min. These results indicated that Tartary buckwheat extracts and rutin can inhibit the formation of HMF in the simulated system. The rutin group had the largest reduction efficiency on HMF content, followed by TBS treatment group, but there was no noticeable difference between the TB extracts group and rutin group (*P* > 0.05). In the simulated system, Tartary buckwheat extract demonstrated a significant reduction in HMF levels, and the inhibitory effect closely related to the trend observed in the regulation of AA content. This finding concluded that the extracts and rutin can inhibit AA through decreasing the formation of HMF. Present findings are consistent with previous reports. HMF in buckwheat flour bread and wheat flour bread with quercetin were lower than that in wheat flour bread without quercetin, and the presence of 1.90 mg/g quercetin decreases the HMF content by 69.9 % (Zhang & An, 2017). They further confirmed that quercetin traps HMF or its precursors to form adducts. Epigallocatechin gallate, epigallocatechin, epicatechin and gallic acid significantly inhibited 5-HMF formation in thermal model reaction (Wang et al., 2023). Qi et al. (2018) considered that the condensation of epicatechin and HMF was one of the key steps leading to the inhibition of acrylamide. In addition, Cai et al. (2014) found that chlorogenic acid increased AA formation by enhancing HMF, but its quinone derivative significantly reduced HMF formation.

### 3.8 Effect of extracts on oxidation-reduction potential in the Maillard reaction system

The kinetic results indicate that the formation of AA in food during the Maillard reaction is a dynamic process of simultaneous formation and elimination. AA content significantly decreased after long-term heat treatment (Hedegaard et al., 2008), which indicating that the elimination rate of acrylamide is higher than the formation rate in the final stage of the reaction. Ou et al. (2010) proposed that the antioxidants destructed AA in two ways: attacking the alkene bond of AA by formation of free radicals and directly reacting with acrylamide by the oxidized antioxidant. The change of free radicals can be reflected by the change of oxidation-reduction potential (ORP) of the system. Therefore, ORP changes during the Maillard reaction were determined. Fig. 5 (C) showed that the ORP of the reaction system increased with the extension of heating time in all groups. The ORP of reaction system treated with extracts or rutin was significantly greater than that of control group. The increased efficiency of rutin was strongest at reaction time for 25–30 min, but before 25 min, the efficiency of rutin was lower than that of TBS extracts. The findings indicated that Tartary buckwheat extracts and rutin notably enhanced the positive voltages in asparagine-glucose reaction system and promoted rise of ORP (*P* < 0.05). This phenomenon might represent one of the key mechanisms contributing to the reduction of AA levels. Hedegaard et al. (2008) indicated that effect of oxidative processes on AA elimination should not be neglected, because oxidized vegetable oil seems to promote degradation of acrylamide. Therefore, maintaining a high positive voltage in the system can significantly enhance the reaction between AA and free radicals, thereby promoting the degradation of acrylamide. Ou et al. (2010) reported that antioxidants could inhibit AA formation via destructing the formed acrylamide by their oxidized products in high-temperature processing foods.

### 3.9 Effect of extracts on the elimination of AA

**Fig. 6:**
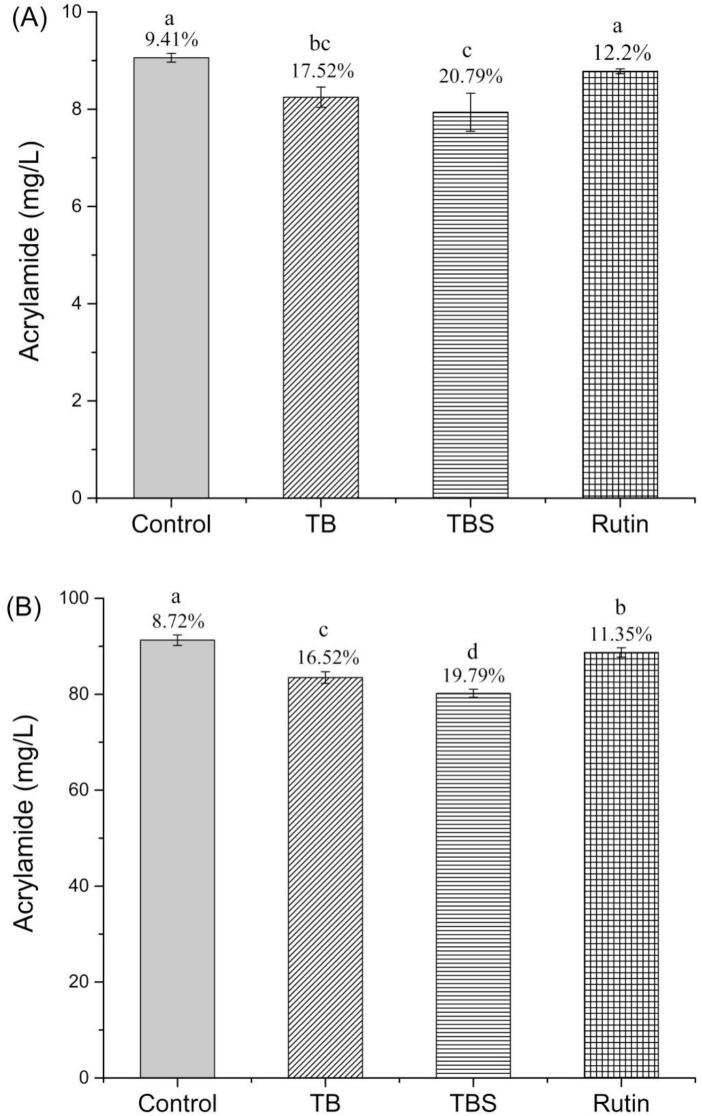
Changes in AA content after AA reaction with TB, TBS extracts, and rutin heated in an oven at 160 °C for 30 min. (A) 10 mg/L acrylamide; (B) 100 mg/L acrylamide. Results are presented as mean ± SD (n = 3). Different letters indicate significant differences (*P* < 0.05).

To further verify the effect of Tartary buckwheat extracts on the elimination of AA, extracts and rutin was added to AA standard (10 mg/L and 100 mg/L) respectively and reacted at 160 °C for 30 min. Fig. 6 (A) showed that the standard solution of AA of 10 mg/L with addition of extracts and rutin after heat treatment contained lower AA levels compared to control group. AA content decreased by 17.52 %, 20.79 % and 12.20 % with addition of extracts of TB, TBS and rutin respectively. The AA content in the control group after heat treatment was reduced by 9.41 %. Meanwhile, similar results were obtained in the standard solution of AA with 100 mg/L. As showed in Fig. 6 (B), AA content decreased by 16.52 %, 19.79 % and 11.35 % with addition of TB, TBS extracts and rutin respectively, while the AA content in the control group was reduced by 8.72 %. These results showed that both TB and TBS extracts significantly promoted the elimination of AA at both low and high AA concentrations of the AA (*P* < 0.05). Present results are consistent with previous findings. Ou et al. (2008) reported that catechin (15 mg/mL) could eliminate 10.8 % of the AA in model system. Liu et al. (2023) indicated that AA prefers to react with quinone via nucleophilic reaction at high temperature, therefore, native or added catechin and its derivative, catechin quinone, can eliminate AA during thermal processing.

## 4 Conclusions

In the asparagine-glucose simulated system, the inhibitory rate of Tartary buckwheat extracts on AA formation reached the maximum when the concentration of extracts was 1 mg/mL, and TBS extract had the highest inhibition rate on AA formation. Logistic-Exponential kinetic model was most suitable for describing the kinetic behavior of AA in this study. The kinetic parameter *k*_f_ and *t*_f_ of TBS treatment group and rutin treatment group were markedly different from that of control group (*P* < 0.05), which suggested that addition of TBS extract and its main polyphenol component (rutin) could markedly reduce the rate of AA production (*P* < 0.05) and prolong the reaction time when the AA levels reached the maximum values. Three reaction procedures could be involved in Tartary buckwheat extracts and rutin for reducing AA formation. Firstly, Tartary buckwheat extracts and rutin decreased the conversion of 3-APA to AA. Secondly, they reduced the formation of HMF. Thirdly, they enhanced the elimination of AA by increasing the positive voltage of the model system, which promoted the oxidation of AA and the reaction of AA with free radicals.

## CRediT authorship contribution statement

**Wen Zhao:** Writing – original draft, Visualization, Methodology, Investigation, Formal analysis, Data curation, Conceptualization. **Yuchun Jing:** Writing – original draft, Visualization, Validation, Methodology, Investigation, Data curation, Conceptualization. **Xiaoping Li:** Writing – review & editing, Validation, Supervision, Project administration, Methodology, Investigation, Funding acquisition, Conceptualization. **Xinzhong Hu:** Resources, Funding acquisition. **Lixia Wang:** Supervision.

## Declaration of competing interest

The authors declare that they have no known competing financial interests or personal relationships that could have appeared to influence the work reported in this paper.

## Data Availability

The original contributions presented in this study are included in this article. Further inquiries can be directed to the corresponding author.

## References

[bb0005] Bhinder S., Singh N., Kaur A. (2022). Impact of germination on nutraceutical, functional and gluten free muffin making properties of Tartary buckwheat (*Fagopyrum tataricum*). Food Hydrocolloids.

[bb0010] Cai Y., Zhang Z.H., Jiang S.S., Yu M., Huang C.H., Qiu R.X., Li Y.Q. (2014). Chlorogenic acid increased acrylamide formation through promotion of HMF formation and 3-aminopropionamide deamination. Journal of Hazardous Materials.

[bb0015] Chaaban H., Ioannou I., Chebil L., Slimane M., Gérardin C., Paris C., Charbonnel C., Chekir L., Ghoul M. (2017). Effect of heat processing on thermal stability and antioxidant activity of six flavonoids. Journal of Food Processing and Preservation.

[bb0020] Chen Y., Zhu Y., Qin L.K. (2022). The cause of germination increases the phenolic compound contents of Tartary buckwheat *(Fagorpyrum tataricum)*. Journal of Future Foods.

[bb0025] Cheng J., Chen X.Y., Zhao S., Zhang Y. (2015). Antioxidant-capacity-based models for the prediction of acrylamide reduction by flavonoids. Food Chemistry.

[bb0030] Cheng J., Ren Y.P., Zhang Y., Zhang Y. (2013). Effect of flavonol on reduction of acrylamide in Maillard reaction and its correlation with antioxidant capacity. Journal of Zhejiang University. Agriculture and Life Sciences.

[bb0035] Cheng K.W., Zeng X.H., Tang Y.S., Wu J.J., Liu Z.W., Sze K.H., Wang M.F. (2009). Inhibitory mechanism of Naringenin against carcinogenic acrylamide formation and nonenzymatic Browning in Maillard model reactions. Chemical Research in Toxicology.

[bb0040] Corradini M.G., Peleg M. (2006). Linear and non-linear kinetics in the synthesis and degradation of acrylamide in foods and model systems. Critical Reviews in Food Science and Nutrition.

[bb0045] Crawford L.M., Kahlon T.S., Chiu M.C.M., Wang S.C., Friedman M. (2019). Acrylamide content of experimental and commercial flatbreads. Journal of Food Science.

[bb0050] Gokmen V., Kocadagli T., Goncuoglu N., Mogol B.A. (2012). Model studies on the role of 5-hydroxymethyl-2-furfural in acrylamide formation from asparagine. Food Chemistry.

[bb0055] Hedegaard R.V., Granby K., Frandsen H., Thygesen J., Skibsted L.H. (2008). Acrylamide in bread. Effect of prooxidants and antioxidants. European Food Research and Technology.

[bb0060] Iriondo-DeHond A., Elizondo A.S., Iriondo-DeHond M., Ríos M.B., Mufari R., Mendiola J.A., del Castillo M.D. (2020). Assessment of healthy and harmful Maillard reaction products in a novel coffee cascara beverage: Melanoidins and acrylamide. Foods.

[bb0065] Jing Y.C., Li X.P., Hu X.Z., Ma Z., Liu L., Ma X. (2019). Effect of buckwheat extracts on acrylamide formation and the quality of bread. Journal of the Science of Food and Agriculture.

[bb0070] Ke J., Ran B., Sun P.Y., Cheng Y.Z., Chen Q.F., Li H.Y. (2023). An evaluation of the absolute content of flavonoids and the identification of their relationship with the flavonoid biosynthesis genes in Tartary buckwheat seeds. Agronomy-Basel.

[bb0075] Kim S.J., Zaidul I.S.M., Maeda T., Suzuki T., Hashimoto N., Takigawa S., Yamauchi H. (2007). A time-course study of flavonoids in the sprouts of tartary (*Fagopyrum tataricum* Gaertn.) buckwheats. Scientia Horticulturae.

[bb0080] Knol J.J., Van Loon W.A.M., Linssen J.P.H., Ruck A.L., Van Boekel M.A.J.S., Voragen A.G.J. (2005). Toward a kinetic model for acrylamide formation in a glucose-asparagine reaction system. Journal of Agricultural and Food Chemistry.

[bb0085] Koyama M., Nakamura C., Nakamura K. (2013). Changes in phenols contents from buckwheat sprouts during growth stage. Journal of Food Science and Technology-Mysore.

[bb0090] Ling A.J., Li X.P., Hu X.Z., Ma Z., Wu K.M., Zhang H.W., Wei S.F. (2018). Dynamic changes in polyphenol compounds, antioxidant activity, and PAL gene expression in different tissues of buckwheat during germination. Journal of the Science of Food and Agriculture.

[bb0095] Liu X.Y., Su J.J., Geng Y.Q., Chen F., Huang B.Y., Yang H.J., Ma L.J. (2023). A method on acrylamide elimination: Comparing and tracing reaction pathways of acrylamide and catechin (catechin quinone) using UHPLC-Q-exactive orbitrap mass spectrometry. Food Chemistry.

[bb0100] Liu Y.B., Wang P.P., Chen F., Yuan Y., Zhu Y.C., Yan H.Y., Hu X.S. (2015). Role of plant polyphenols in acrylamide formation and elimination. Food Chemistry.

[bb0105] Maan A.A., Anjum M.A., Khan M.K.I., Nazir A., Saeed F., Afzaal M., Aadil R.M. (2022). Acrylamide formation and different mitigation strategies during food processing - a review. Food Reviews International.

[bb0110] Morales G., Jimenez M., Garcia O., Mendoza M.R., Beristain C.I. (2014). Effect of natural extracts on the formation of acrylamide in fried potatoes. LWT-Food Science and Technology.

[bb0115] Mottram D.S., Wedzicha B.L., Dodson A.T. (2002). Acrylamide is formed in the Maillard reaction. Nature.

[bb0120] Nagpal T., Alam S., Khare S.K., Satya S., Chaturvedi S., Sahu J.K. (2022). Effect of *Psidium guajava* leaves extracts on thermo-lipid oxidation and Maillard pathway born food toxicant acrylamide in Indian staple food. Journal of Food Science and Technology-Mysore.

[bb0125] Nan X.P., Nan S.L., Zeng X.P., Kang L.N., Liu X.Y., Dai Y.G. (2021). Inhibition kinetics and mechanism of glutathione and quercetin on acrylamide in the low-moisture Maillard systems. Journal of Food Protection.

[bb0130] Oral R.A., Dogan M., Sarioglu K. (2014). Effects of certain polyphenols and extracts on furans and acrylamide formation in model system, and total furans during storage. Food Chemistry.

[bb0135] Ou S.Y., Lin Q., Zhang Y.P., Huang C.H., Sun X., Fu L. (2008). Reduction of acrylamide formation by selected agents in fried potato crisps on industrial scale. Innovative Food Science & Emerging Technologies.

[bb0140] Ou S.Y., Shi J.J., Huang C.H., Zhang G.W., Teng J.W., Jiang Y., Yang B.Y. (2010). Effect of antioxidants on elimination and formation of acrylamide in model reaction systems. Journal of Hazardous Materials.

[bb0145] Pesce F., Ponzo V., Mazzitelli D., Varetto P., Bo S.M.A., Saguy I.S. (2024). Strategies to reduce acrylamide formation during food processing focusing on cereals, children and toddler consumption: A review. Food Reviews International.

[bb0150] Qi Y.J., Cheng J.H., Ding W.M., Wang L., Qian H.F., Qi X.G., Zhang H. (2024). Epicatechin-promoted formation of acrylamide from 3-Aminopropionamide via Postoxidative reaction of B-ring. Journal of Agricultural and Food Chemistry..

[bb0155] Qi Y.J., Zhang H., Zhang H., Wu G.C., Wang L., Qian H.F., Qi X.G. (2018). Epicatechin adducting with 5-Hydroxymethylfurfural as an inhibitory mechanism against acrylamide formation in Maillard reactions. Journal of Agricultural and Food Chemistry.

[bb0160] Ren S.C., Sun J.T. (2014). Changes in phenolic content, phenylalanine ammonia-lyase (PAL) activity, and antioxidant capacity of two buckwheat sprouts in relation to germination. Journal of Functional Foods.

[bb0165] Rizzi G.P. (2003). Electrochemical study of the Maillard reaction. Journal of Agricultural and Food Chemistry.

[bb0170] Sarion C., Codina G.G., Dabija A. (2021). Acrylamide in bakery products: A review on health risks, legal regulations and strategies to reduce its formation. International Journal of Environmental Research and Public Health.

[bb0175] Shi J.Q., Shao Z.P., Li H.L., Zhang Y., Wang S. (2019). Co-extraction and co-purification coupled with HPLC-DAD for simultaneous detection of acrylamide and 5-hydroxymethyl-2-furfural in thermally processed foods. Molecules.

[bb0180] Uthra C., Reshi M.S., Jaswal A., Yadav D., Shrivastava S., Sinha N., Shukla S. (2022). Protective efficacy of rutin against acrylamide-induced oxidative stress, biochemical alterations and histopathological lesions in rats. Toxicology Research.

[bb0185] Verma V., Yadav N. (2024). Effect of plant extracts on the reduction of acrylamide and hydroxymethylfurfural formation in French fries. Food Chemistry Advances.

[bb0190] Wang W.T., Wang H.X., Wu Z.J., Duan T.T., Liu P.Z., Ou S.Y., Zheng J. (2023). Reduction in five harmful substances in fried potato chips by pre-soaking treatment with different tea extracts. Foods.

[bb0195] Wu H.J., Zheng J., Zhang G.W., Huang C.H., Ou S.Y. (2018). The formation of acrylamide from and its reduction by 3-Aminopropanamide occur simultaneously during thermal treatment. Journal of Food Science.

[bb0200] Xu X., An X.N. (2016). Study on acrylamide inhibitory mechanism in Maillard model reaction: Effect of p-coumaric acid. Food Research International.

[bb0205] Xue C.Y., Li Y., Quan W., Deng P., He Z.Y., Qin F., Zeng M.M. (2022). Unraveling inhibitory effects of *Alpinia officinarum Hance* and curcumin on methylimidazole and acrylamide in cookies and possible pathways revealed by electron paramagnetic resonance. Food Chemistry.

[bb0210] Yu S.J., Chen Z.J., Meng H.C., Chen M.S. (2020). Addition of lipophilic grape seed proanthocyanidin effectively reduces acrylamide formation. Journal of the Science of Food and Agriculture.

[bb0215] Zamora R., Brenes-Alvarez M., Hidalgo F.J. (2025). Formation of acrylamide in the presence of quinones. Food Chemistry.

[bb0220] Zhang M., Chen H.X., Li J.L., Pei Y., Liang Y. (2010). Antioxidant properties of tartary buckwheat extracts as affected by different thermal processing methods. LWT-Food Science and Technology.

[bb0225] Zhang T., Zhang C.M., Luo Y.Y., Liu S.P., Li S.Y., Li L.X., Liu J. (2023). Protective effect of rutin on spinal motor neuron in rats exposed to acrylamide and the underlying mechanism. Neurotoxicology.

[bb0230] Zhang Y., Wang Q., Huang M.M., Chen X.Y. (2015). Unravelling the effect of flavonoids on the kinetic profiles of acrylamide in the Maillard reaction. RSC Advances.

[bb0235] Zhang Y., Zhang Y. (2008). Effect of natural antioxidants on kinetic behavior of acrylamide formation and elimination in low-moisture asparagine-glucose model system. Journal of Food Engineering.

[bb0240] Zhang Y.N., An X.N. (2017). Inhibitory mechanism of quercetin against the formation of 5-(hydroxymethyl)-2-furaldehyde in buckwheat flour bread by ultra-performance liquid chromatography coupled with high-resolution tandem mass spectrometry. Food Research International.

[bb0245] Zhu F., Cai Y.Z., Ke J.X., Corke H. (2009). Evaluation of the effect of plant extracts and phenolic compounds on reduction of acrylamide in an asparagine/glucose model system by RP-HPLC-DAD. Journal of the Science of Food and Agriculture.

[bb0250] Zhu Y.C., Luo Y.H., Sun G.Y., Wang P.P., Hu X.S., Chen F. (2022). The simultaneous inhibition of histidine on 5-hydroxymethylfurfural and acrylamide in model systems and cookies. Food Chemistry.

